# Correction: A novel power-amplified jumping behavior in larval beetles (Coleoptera: Laemophloeidae)

**DOI:** 10.1371/journal.pone.0304024

**Published:** 2024-05-16

**Authors:** Matthew A. Bertone, Joshua C. Gibson, Ainsley E. Seago, Takahiro Yoshida, Adrian A. Smith

The images for Figs 2 and 3 are incorrectly switched. The image that appears as Fig 2 should be Fig 3, and the image that appears as Fig 3 should be Fig 2. The figure captions appear in the correct order and please see the correct Figs 2 and 3 here.

**Fig 2:**
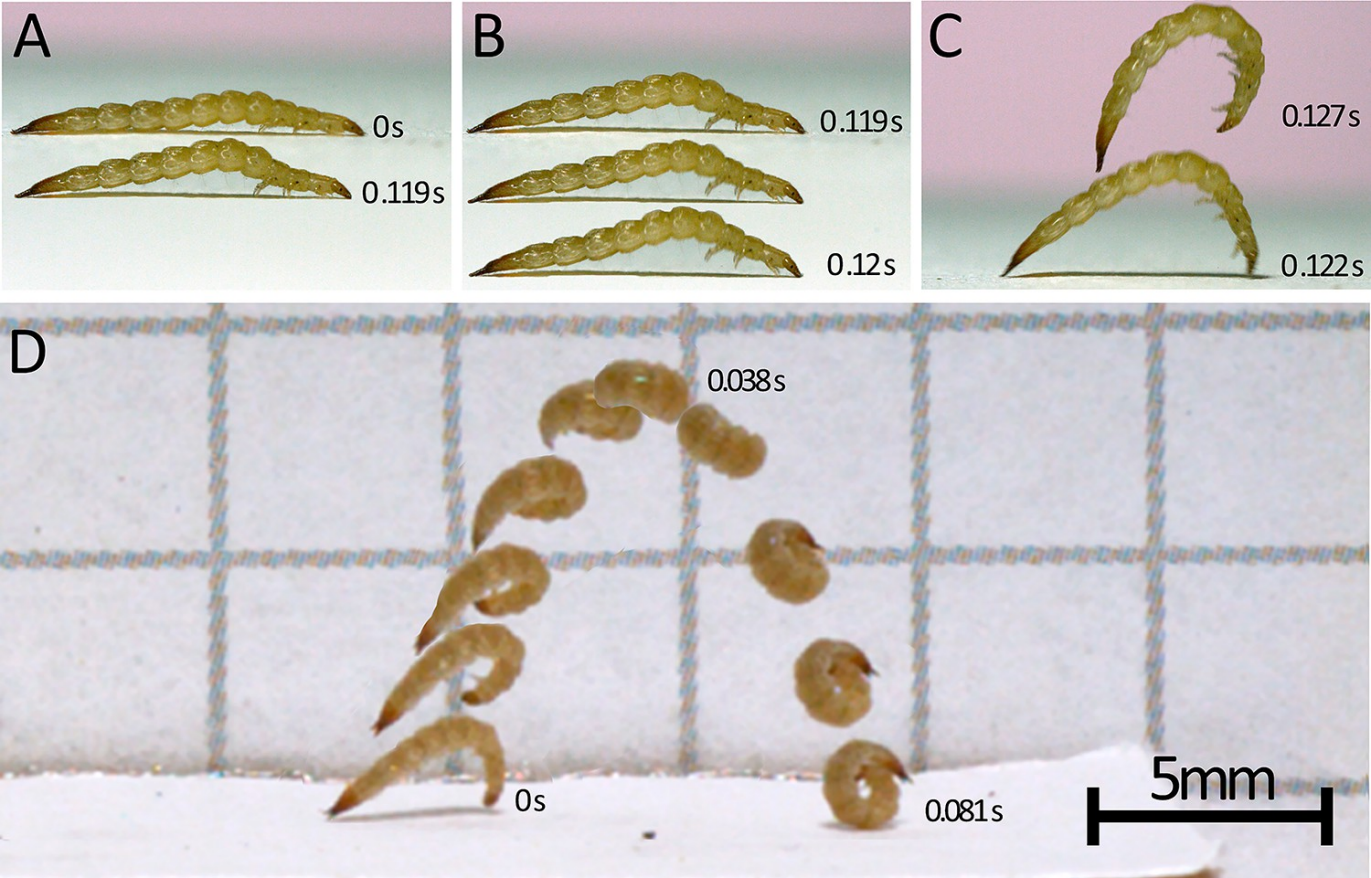
Laemophloeus biguttatus jump sequences (A-C and D are separate jumps) taken from videos filmed at 3,200 frames per second. A-C: loading, latch-decoupling, and launch phases of a jump, timecode labels on images correspond to the image of the beetle they are nearest; D: complete jump trajectory. A: loading phase, during which the body slowly bends ventrally. 0.119 seconds elapse between top and bottom body postures. Bottom image is the frame directly preceding the top larval image in B. B: latch-decoupling sequence, during which the legs release or lose their grip on the substrate. In the pictured jump sequence, the hind and midlegs are first to release their grip, followed by the forelegs. Each larval image is a single sequential frame and only 0.625 ms separate the top and bottom image. C: launch phase, corresponding to the transfer of stored energy to the kinetic energy of the body moving into the air. Shown here are the last frames of the launch phase depicting the last frame in which the larvae has any contact with the substrate (below) and a frame from the airborne phase (above). 5.31 ms separate the bottom image from the top. The bottom image in panel C is 1.8 ms after the bottom image in panel B. D: Airborne phase of a separate jump from that depicted in panels A-C. The entire sequence spans 0.081 seconds, noted times pertain to the first, top, and last of the sequential images, and the scale bar pertains only to this panel.

**Fig 3:**
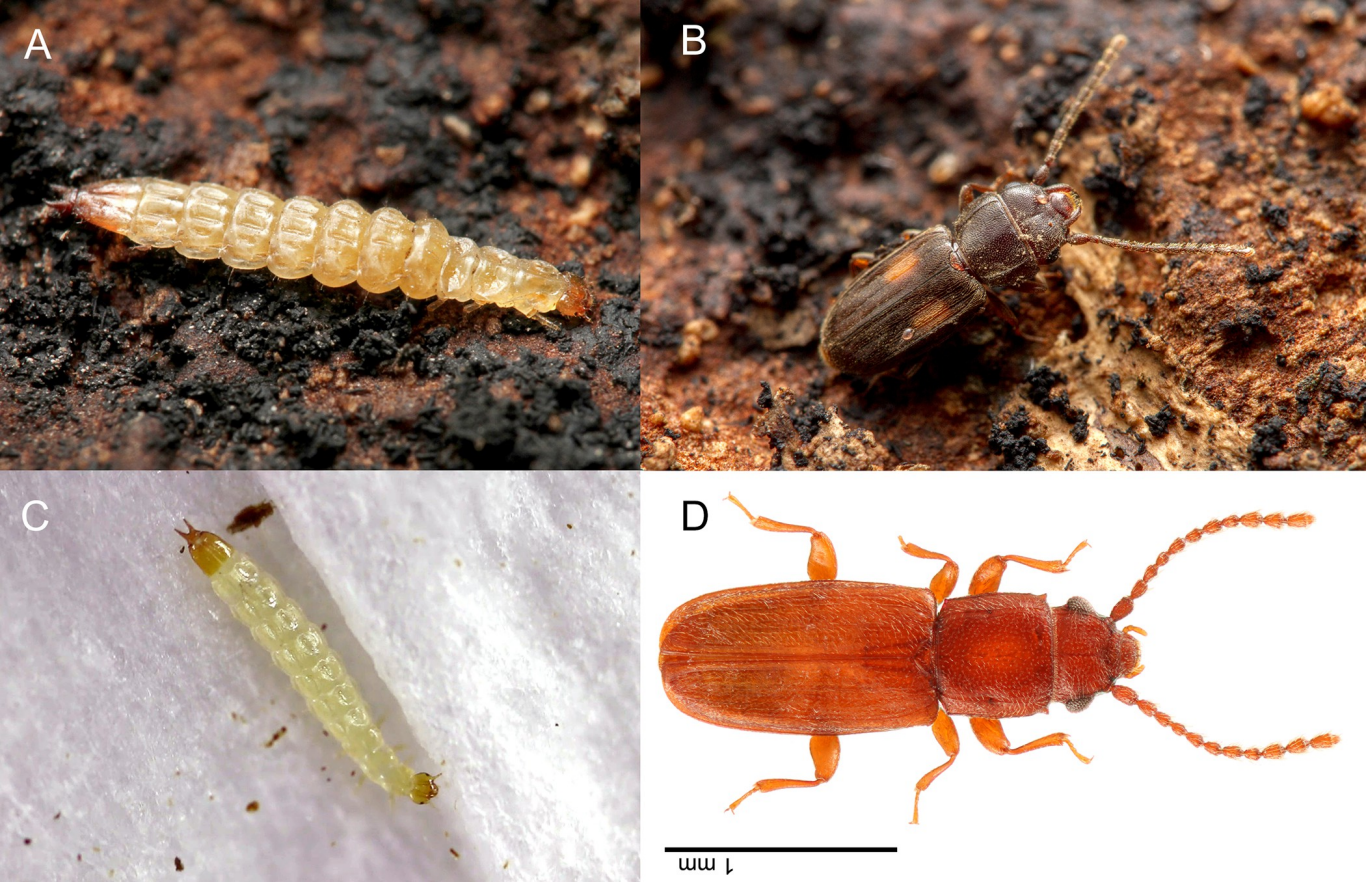
Habitus images of known Laemophloeidae with jumping larvae: A: larva of *Laemophloeus biguttatus*; B: same, adult; C: larva of *Placonotus testaceus*, D: same, adult. (A&B: taken by MAB; D&C: taken by TY).
